# Gross Anatomical Studies on the Hind Limb of the West African Giraffe (*Giraffa camelopardalis peralta*)

**DOI:** 10.1155/2021/8818525

**Published:** 2021-07-03

**Authors:** Kenechukwu T. Onwuama, Sulaiman O. Salami, Esther S. Kigir, Alhaji Z. Jaji

**Affiliations:** Department of Veterinary Anatomy, University of Ilorin, Ilorin, Nigeria

## Abstract

This study on the gross anatomy of the West African giraffe's hind limb was aimed at investigating the unique morphological features and number of bones making up this region of the skeleton. Two (2) adults obtained as carcasses at different times after postmortem examination were prepared to extract the bones via cold water maceration for use in the study. The appearance of the ossa coxarum and its features presented similarities to that of the horse. However, differences were evident in the convex cranial border of the ilium, small less prominent coxal tuber, and wider interval between the opposite sacral tuber and an oval obturator foramen. Common features reported in most species such as the gluteal line and psoas tubercle were absent. The long femur presented proximally; the greater trochanter connected obliquely via the trochanteric crest to the lesser trochanter. The supracondyloid fossa, obliquely directed medial condyle, and trochlea with two unequal ridges were presented distally. The fibula was absent while the tibia was typical of ruminant presentation with one popliteal line and no muscular lines on its caudal surface. The five (5) tarsals were arranged three proximally and two distally. One (1) metatarsal (3rd and 4th fused) presented two condyles that anchor two (2) digits with 3 phalanges and 3 sesamoid bones each. The total number of bones making up the hind limb was accounted to be 45. In conclusion, this study has provided a baseline data for further biological, archeological, and comparative anatomical studies.

## 1. Introduction

The West African giraffe (*Giraffa camelopardalis*), the tallest living terrestrial mammal and largest ruminant, is a representative of the Graffidae family of the order Artiodactyla consisting of one species having multiple subspecies [1, 2]. It is a native of Africa, especially the sub-Saharan regions with their distribution including several fragmented parts of the continent [3].

It has however been extirpated from much of their historical range due to different parts of their body being used for different purposes [4], therefore being listed as vulnerable [5, 6] with some subspecies even endangered [7]. Though still found in numerous national parks and game reserves with a few in the wild [8], its dwindling population has raised concerns for its protection and further domestication in private game reserves and zoos [9]. Although studies on the various physiological, behavioural, and biological aspects of this mammal have been reported, none has been documented on the detailed description of the bones that make up its skeleton. To this end, this study on the bones of the hind limb has been conducted to investigate normal gross features and number making up this region. It also serves as a foundation for comparative study to differentiate it from food animals and the horse.

## 2. Materials and Methods

Two (male and female) West African giraffe (*Giraffa camelopardalis peralta*) carcasses weighing 1,070 kg and 812 kg were obtained after postmortem from the Department of Veterinary Pathology, University of Ilorin at different periods of time. This small sample size is permitted and quite understandable given that giraffe carcasses are not commonly available for scientific research. They were transported to the Veterinary Anatomy gross laboratory, University of Ilorin, Nigeria, for bone preparation as museum specimens. They were carefully defleshed using sharp knife and scalpel blades. The skin was removed, and muscles teased out, leaving the bones with minimal soft tissue attachment before being transferred to a large container of cold water enough to submerge the bones at room temperature. The container was covered airtight and placed away from the sun throughout the period of maceration with regular change of water. After completion, the bones were recovered after draining, and sun dried. Photographs of recovered bones were taken individually. They were also articulated using glue, noting the bones that constituted the pes of the hind limb.

## 3. Results

The hind limb comprised of bones of the hip (ossa coxarum), thigh (femur), leg (tibia and fibula), and pes (tarsals, metatarsals, and digits). They exhibited general and peculiar features similar to and different from the bovids and equids. The average number of bones of the hind limb was found to be 45 as shown in Table 1.

**Figure 1:**
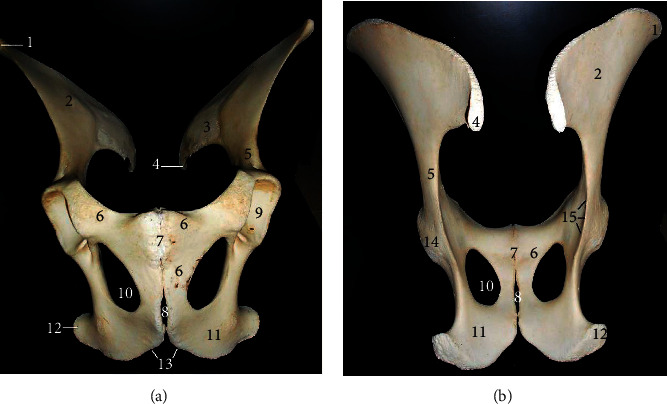
Ossa coxarum of the West African giraffe (*Giraffa camelopardalis peralta*). Ventral (a) and dorsal (b) views. (1) Coxal tuber; (2) wing of ilium (quadrilateral surface); (3) auricular surface of wing; (4) sacral tuber; (5) body of ilium; (6) pubis; (7) pubic symphysis; (8) ischial symphysis; (9) cotyloid cavity; (10) obturator foramen; (11) ischium; (12) ischial tuber; (13) ischial arch; (14) acetabulum; and (15) ischiatic spine.

The ossa coxarum presented two halves joined at the pelvic symphysis (Figure 1). It consisted of 3 fused bones; ilium cranially, ischium caudally, and pubis medially all presenting parts that formed the acetabulum for articulation with the head of femur. The *ilium* presented a wing cranially and a body caudally that extended to form the cranial part of the acetabulum. The wing presented two surfaces (dorsal and ventral) with a convex cranial border, concave medial, and lateral borders. It formed the sacral and coxal tubers at its medial and lateral angles, respectively. The dorsolateral surface was smooth while the ventral surface presented an inner auricular area (for sacrum articulation) and an outer quadrilateral area. On the ventral surface of the body cranial to the acetabulum, it presents a fossa (Figure 1). The pubis formed the craniomedial aspect of the bone extending laterally (to form the medial part of the acetabulum) and caudally (to join the ischium). Its cranial border presented pubic eminences that flanked the pubic symphysis. Its caudal border formed the cranial margin of the large oval obturator foramen. The ischium formed the caudolateral aspect of the bone. Its craniolateral angle formed the caudal part of the acetabulum, and its caudolateral angle formed the ischial tuber while the caudomedial aspect joined its opposite to form a V-shaped ischiatic arch. The cranial border forms the caudal margin of the obturator foramen. The acetabulum presented a deep cotyloid cavity that was notched caudally (Figure 1).

**Figure 2:**
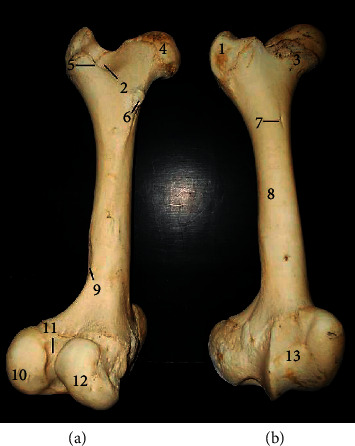
Femur of the West African giraffe (*Giraffa camelopardalis peralta*). Caudal (a) and cranial (b) views. (1) Greater trochanter; (2) trochanteric fossa; (3) neck; (4) head; (5) trochanteric crest; (6) lesser trochanter; (7) nutrient foramen; (8) shaft; (9) supracondyloid fossa; (10) lateral condyle, (11) intercondylar fossa; (12) medial condyle; and (13) trochlea.

**Figure 3:**
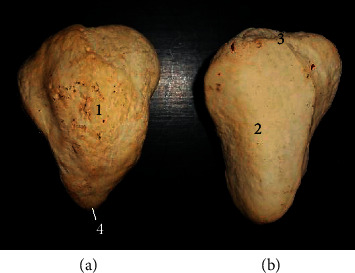
Patella of the West African giraffe (*Giraffa camelopardalis peralta*). Cranial (a) and caudal (b) views. (1) External surface; (2) internal or articular surface; (3) base; and (4) apex.

The femur (Figure 2) presented a long bone with a shaft and two extremities (proximal and distal). The shaft presented a convex cranial surface with a nutrient foramen at its proximal third while the concave caudal surface presented the lesser trochanter on the proximal third of its medial surface. The supracondyloid fossa was visible on the distal third of the caudolateral surface. The proximal extremity presented the head and neck medially and greater trochanter laterally. A curved trochanteric crest connected the greater trochanter obliquely to the lesser trochanter while creating the trochanteric fossa. The distal extremity presented caudally, the lateral condyle, and obliquely directed medial condyle separated by the intercondylar fossa. Cranially, it presented a trochlea with two unequal ridges. The triangular patella (Figure 3) presented a convex dorsal base, ventral apex, concave inner articular surface, and convex roughened external surface.

**Figure 4:**
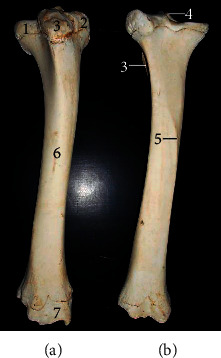
Tibia of the West African giraffe (*Giraffa camelopardalis peralta*). Cranial (a) and caudal (b) views. (1) Lateral condyle; (2) medial condyle; (3) tibial tuberosity; (4) intercondyloid eminence; (5) popliteal line; (6) shaft; and (7) medial malleolus.

**Figure 5:**
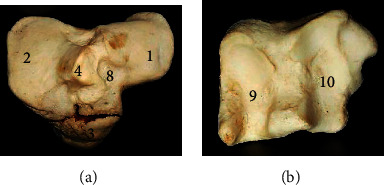
Articular surfaces of proximal (a) and distal (b) extremities of the tibia. (1) Lateral condyle; (2) medial condyle; (3) tibia tuberosity; (4) intercondyloid eminence; (8) intercondyloid fossa; and (9) and (10) articular grooves for tibiotarsal articulation.

The longest bone tibia (Figure 4) presented a shaft and two extremities (proximal and distal). The shaft had a slightly twisted appearance having three surfaces at the proximal third (lateral, medial, and caudal) and two surfaces on its distal third (cranial and caudal). Its caudal surface presented one popliteal line running obliquely at the midshaft. The proximal extremity (Figure 5) bore the lateral, medial condyles (separated by a fossa), and cranial tibia tuberosity. The medial condyle extended centrally to form the intercondyloid eminence. The distal extremity (Figure 5) presented an articular surface marked with depressions for articulation with the tibia tarsal bone. It extended the medial malleolus. The fibula was absent in this species while the lateral malleolus was formed by a small irregular malleoli bone.

**Figure 6:**
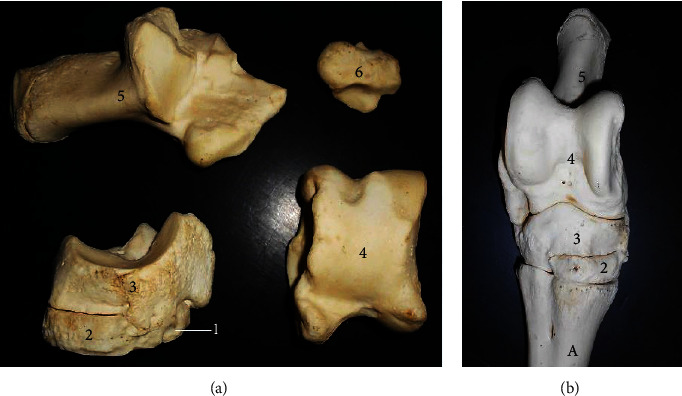
Tarsals and malleolus of the West African giraffe (*Giraffa camelopardalis peralta*). Dorsal view (b). (1) First tarsal; (2) second and third tarsal fused; (3) centriquartal (central and fourth tarsal fused); (4) talus; (5) calcaneus; and (6) os malleolus.

The tarsals (Figure 6) were five (5) in number, namely, fibula tarsal, tibia tarsal, first tarsal, and second and third tarsal fused and centriquartal. The fibula tarsal (calcaneus) presented the calcaneal tuber dorsally and the sustentacular tali medially. The tibia tarsal (talus) presented proximal and distal condyles that were separated by a groove.

**Figure 7:**
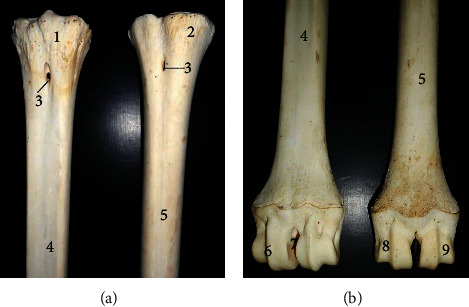
Proximal and distal halves of metatarsal bones of the left and right hind limb, respectively, of the West African giraffe (*Giraffa camelopardalis peralta*), volar (a) and dorsal (b) views. (1) Proximal extremity; (2) head; (3) metatarsal canal; (4) metacarpal groove; (5) shaft; (6) median ridge; (7) intercondylar groove; (8) medial condyle; and (9) lateral condyle.

The metatarsals (Figure 7) presented long bones with shaft and two extremities (proximal and distal). A shallow longitudinal groove spanned the shaft length of the volar surface and proximal one-third of the dorsal surface. The metatarsal canal was only seen proximally. The proximal extremity presented articular surfaces for tarsal articulation and its dorsal surface roughened for muscle attachment. The distal extremity comprised lateral and medial condyle separated by an intercondylar groove. Each condyle presented a median ridge.

**Figure 8:**
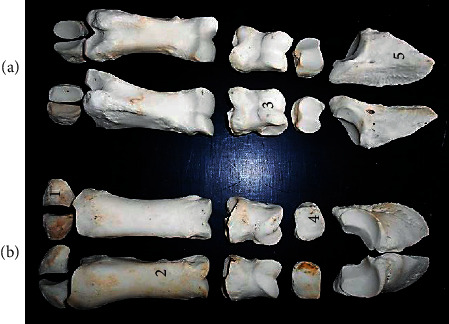
Left and right hind limb digits of the West African giraffe (*Giraffa camelopardalis peralta*), volar (a) and dorsal (b) views. (1) Proximal sesamoid; (2) proximal phalanx; (3) middle phalanx; (4) distal sesamoid; and (5) distal phalanx.

Two digits (Figure 8) having three phalanges were seen on each foot. The first and second phalanges were small long bones with shaft and extremities that presented concave proximal surface and convex distal surfaces for articulation. The second phalanx was the shortest of the three bones. The third phalanx presented a long triangular shaped bone with a concave surface at its craniodorsal end (for articulation with the second phalanx) and lateral, medial, and ventral surfaces. Six sesamoid bones were found on the palmar surface of each foot with two located at the metatarsophalangeal joint and one at the distal interphalangeal joint of each digit.

## 4. Discussion

Bones of the West African giraffe hind limb presented unique morphological features in comparison to the bovids and equids previously studied. Despite being the largest ruminant, the appearance of the ossa coxarum together with specific features such as the upward curved sacral tuber, single notched acetabulum, V-shaped ischial arch, and an undivided sacral tuber presented similarities to that of the horse [10]. However, differences were evident in its convex cranial border of the ilium, small less prominent coxal tuber, and wider interval between the opposite sacral tuber and an oval obturator foramen. Common features reported in the hip bones of most species such as the gluteal line and psoas tubercle were absent in this species.

The femur's appearance as a long bone with features such as the greater trochanter connecting obliquely via the trochanteric crest to the lesser trochanter; shallow trochanteric fossa, distal supracondyloid fossa, and an obliquely directed medial condyle and trochlea with two unequal ridges were typical of presentations reported in the goat, ox and pig [11, 12].

The tibia was also typical of ruminant presentation except that the muscular lines expected on the caudal surface were not visible while only a single popliteal line was present. The fibula was also absent although a separate bone, the os malleolus was seen representing the lateral malleolus unlike some breeds of ox [13] and horse [14] with rudimentary and incomplete fibula, respectively.

The irregular shaped morphology, number, and arrangement (in the live animal) of the tarsal bones were similar to the those reported in other ruminants [15]. The shallow longitudinal groove spanning the shaft length of the volar surface and proximal one-third of the dorsal surface of the metatarsal with its two distal condyles (lateral and medial) were the basis for assumption that the metatarsal was formed from the fusion of the 3rd and 4th bones. This presentation was similar to the ruminants though the longitudinal groove is deeper in those species while the ox (some breeds) has the rudimentary 5th metatarsal [16]. The metatarsal canal was only seen proximally in this species unlike other ruminant species with proximal and distal canals. Also, the morphology, number, and arrangement of the phalanges and sesamoids were similar to that reported in the ox, sheep, and goat [13, 17].

## 5. Conclusion

This study on the gross anatomy of the hind limb of the West African giraffe (*Giraffa camelopardalis peralta)* presented the numerical and morphological information on bones of this animal skeleton highlighting specific features, similarities, and differences from other ruminant and domesticated animals.

## Figures and Tables

**Table 1:** Number of bones of the hind limb of the West African giraffe (*Giraffa camelopardalis peralta*).

Bones	Number
Ossa coxarum	1
Femur	2
Patella	2
Tibia	2
Os malleolus	2
Tarsals	10
Metatarsals	2
First phalanx	4
Second phalanx	4
Third phalanx	4
Sesamoid	12
Total average	45

## Data Availability

Data sharing is not applicable to this article as no datasets were generated or analysed during the current study.
